# Can the rigid stylet from an intravenous catheter be an alternative vascular access device for intraosseous catheterisation?

**DOI:** 10.1002/vms3.1361

**Published:** 2024-01-27

**Authors:** Janghwan Kim, Daesik Kim, Daeyun Seo, Hyejin Hwang, Yuna Kim, Taekyu Chung, Seongsoo Lim, Hansol Lee, Beomkwan Namgoong, Ahreum Choe, Hyeajeong Hong, Heesung Umh, Min Su Kim

**Affiliations:** ^1^ Veterinary Emergency Medicine, Department of Veterinary Clinical Science, College of Veterinary Medicine and Research Institute for Veterinary Science Seoul National University Seoul Republic of Korea

**Keywords:** dog, emergency, intraosseous catheterisation, intravenous catheter, vascular access

## Abstract

**Background:**

It may be challenging for practitioners to secure vascular access in animals, especially in a state of shock.

**Aim:**

This study aimed to evaluate the clinical relevance of commercial 16‐gauge intravenous (IV) catheter stylet vs. 15‐gauge intraosseous (IO) vascular access in emergencies.

**Methods:**

Six healthy dogs were used in this study. After general anaesthesia, IO catheterisation was performed on both trochanteric fossae of the femurs using a commercial 16‐gauge IV catheter stylet and EZIO 15‐gauge IO needle. The pressures were measured by manual, gravity and mechanical infusion for 5 min each. Additionally, insertion time and success rate were recorded.

**Results:**

In hand infusion, mean pressure of a 16‐gauge IO (605.06 ± 49.13 mmHg) was statistically lower than that of a 15‐gauge EZIO IO catheterisation (718.84 ± 93.09 mmHg). In gravity infusion, there was no significant difference in pressure during injection between the 16‐gauge IV catheter (52.23 ± 14.48 mmHg) and 15‐gauge EZIO IO catheterisation (50.68 ± 11.89 mmHg). In mechanical infusion, mean pressure of the 15‐gauge EZIO IO catheterisation (128.25 ± 50.16 mmHg) was lower than that of the 16‐gauge IV catheter (152.56 ± 67.23 mmHg). The insertion times of a 16‐gauge IV catheter and 15‐gauge EZIO IO catheterisation were 16.79 ± 0.92 and 11.65 ± 1.70 s, respectively. The success rates of the insertion were similar between both groups.

**Conclusion:**

This study shows that IO catheterisation with a commercial IV catheter stylet can be an alternative method of IO catheterisation in an emergency, especially for rapid vascular access.

## Graphical abstract

In this study, we investigated 16‐gauge intravenous (IV) catheters as an alternative to 15‐gauge intraosseous (IO) catheters. Based on our results, we concluded that IO catheterisation using an IV catheter can be an alternative to 15‐gauge EZIO catheterisation.

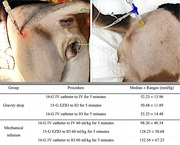

## 1 INTRODUCTION

Securing vascular access in animals is crucial and often lifesaving in emergencies. Most emergencies require rapid procedures, such as injecting drugs or fluid resuscitation. However, it may be challenging for practitioners to place catheters in peripheral vessels, especially in states of shock or any other emergency conditions. The utilisation of intraosseous (IO) catheterisation is not uncommon in veterinary medicine for the administration of fluids or medications when conventional vascular access is not achievable. This method provides a dependable and non‐collapsible means of rapidly accessing the vasculature, ensuring the safe delivery of fluids and drugs, particularly in situations involving pediatric patients, trauma cases, hypovolemia, cardiovascular collapse or shock‐induced hypotension (Lange et al., 2019). One research investigated and compared the time required and success rates between IO catheterisation and jugular venous catheterisation. The median time for all IO catheterisations was 55.4 s (ranging from 15.0 to 153.0 s), while the median time for all jugular venous catheterisations was 217.3 s (ranging from 55.6 to 614 s). Both methods exhibited an equal success rate of 87.5% (Allukian et al., 2017). Furthermore, another research focused on comparing IO catheter placement difficulty, flow rates and success rates at four different locations in canine cadavers. The femur and humerus were found to have high success rates for IO catheterisation, with relatively short placement times and low difficulty scores (Lange et al., 2019). Other studies reported IO catheterisation with an automatic bone injection device (EZIO, Teleflex) facilitates vascular access in dogs and in cat cadavers (Bukoski et al., 2010; Olsen et al., 2002). Many studies suggest that IO catheterisation in animals suffering from cardiovascular collapse could be an alternative method to intravenous (IV) catheterisation. However, the financial burden of using IO catheters requiring automatic injection devices in veterinary medicine may be more significant than expected, and there are limited studies on alternative options for vascular access. A study wherein inexperienced clinicians used a hypodermic needle for IO catheterisation in rabbit cadavers had a moderate success (Kennedy et al., 2020). In humans, the American Heart Association supports the use of IO catheters as a reasonable alternative to IV catheterisation (Blumberg et al., 2008; Frascone et al., 2015). The purpose of this study was to evaluate the potential utility of an IV catheter stylet as an alternative for IO vascular access to replace an EZIO IO vascular access device with a commercial IV catheter for IO vascular access by comparing the infusion pressures. We hypothesise that IV catheter stylets can be used as an alternative to commercial IO access products.

## 2 MATERIALS AND METHODS

### 2.1 Animals

This study was approved by the Institutional Animal Care and Use Committee at Seoul National University (230906‐7‐1). Six neutered female beagle dogs aged 12−18 months, weighing 10–15 kg, were included in the study. All study dogs were clinically normal based on a physical exam, vital signs and blood pressure.

### 2.2 Experimental protocol

All dogs were premedicated with tramadol (Trodon Injection, Aju Pharm Co. Ltd.) 3 mg/kg and medetomidine (Domitor, Orion Pharm) 0.01 mg/kg, both given IV. Anaesthesia was maintained with 2%−3% isoflurane (Ifran Liq., Hana Pharm Co. Ltd.) after IV induction with alfaxalone (Alfaxan, Jurox Inc.) 2 mg/kg. Cefazolin (Cefazoline Injection, Chongkundang) 25 mg/kg was given IV to all the dogs before the procedures. The trochanteric fossa of the femur was chosen as the insertion site. The insertion sites were clipped and aseptically prepared. A 15‐gauge (G) EZIO IO vascular access needle and a 16‐G IV catheter with equal length were selected for comparison in this study. For femoral IO insertion, the 15‐G IO vascular access needle (45 × 1.8 mm, The Arrow EZIO Needle Set, Teleflex) and 16‐G IV catheter stylet without plastic venous cannula (45 × 1.7 mm, BD Angiocath Plus, BD) were used as catheters.

**FIGURE 1:**
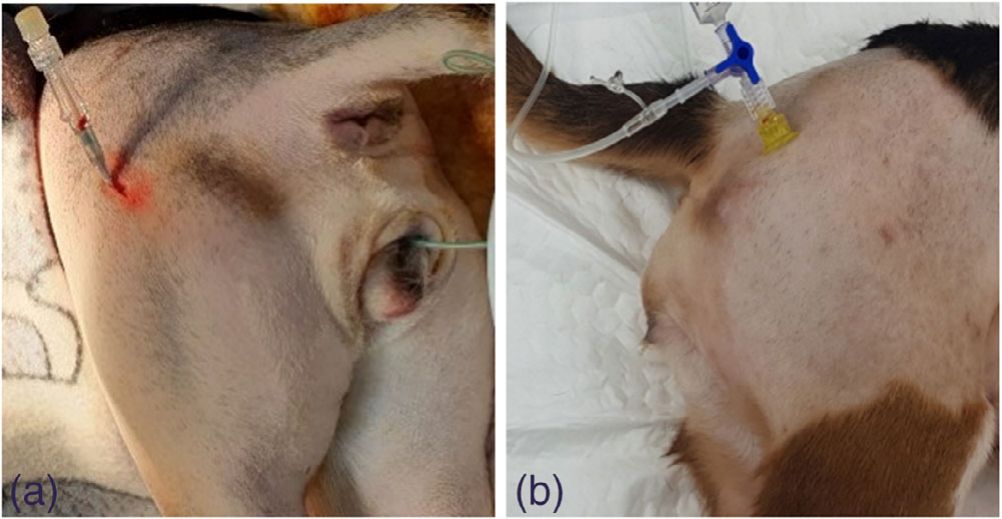
Femoral intraosseous (IO) catheterisation in six beagle dogs receiving IO catheterisation with a standard commercial catheter, compared to an EZIO cannula. IO catheter was placed at the trochanter fossa. 16‐G intravenous (IV) catheter (a), 15‐G EZIO IO needle (b).

**FIGURE 2:**
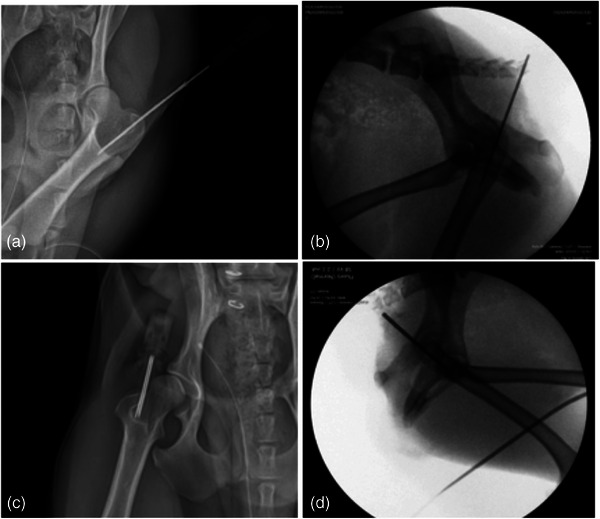
Radiographs and fluoroscopy of the placement in six beagle dogs receiving IO catheterisation with a standard commercial catheter, compared to an EZIO cannula. Radiographic images of 16‐G IV catheter IO insertion (a, b) and 15‐G EZIO IO insertion (c, d).

A power driver (EZIO Power driver, Teleflex) and a commercial electrical drill (Robert Bosch GmbH) were prepared by plasma sterilisation. Animals were placed in lateral recumbency to insert IO needles. Catheters were inserted into the proximal femurs for each dog. IO devices were placed by a single practitioner who had worked in the veterinary teaching hospital and had experience in placing IO catheters. For insertion, the IO needle was placed perpendicular to the trochanteric fossa. IO catheters and IV catheters were placed in the trochanteric fossa of both femurs (Figure 1a,b). An IO vascular needle was inserted by using the power driver, and a standard IV catheter was inserted by using the commercial electrical drill. Procedure time was recorded immediately before the catheters access into the femurs of each dog and stopped after they were believed to be placed successfully by an independent observer. Normal saline (NS) was used for flushing to clear the possible bone fragments in the catheter. Radiography and fluoroscopy were used to verify the placement of both the standard IV catheter and IO needle. (Figure 2a–d). After the insertion, a four‐way stop cock was placed, and a pressure gauge was installed.

### 2.3 Pressure measurement

The pressure of IO catheterisation with EZIO and IV catheters was compared with the pressure of cephalic vein catheterisation as baseline data. A pressure gauge (Fieldlab, Ralston Instruments) was used to measure the pressure during injection, and data were collected by Fieldlab software (Fieldlab Desktop, Ralston Instruments). The pressure was measured as millimetre of mercury (mmHg). The pressure transducer was connected to the middle of the four‐way stop cock and the needle hub to the proximal IO cannula. Pressure was measured under three different circumstances of fluid infusion: (1) 5 mL of NS (0.9% NS) was infused manually for 5 s five times, and maximum pressure was measured; (2) NS was infused by gravity for 5 min, and the average pressure was measured; and (3) NS was infused at 60 mL/kg/h using a mechanical infusion pump (Medifusion DI‐2000, Daiwa Corp. Ltd.) for 5 min, and the average pressure was measured. Each trial proceeded as independent replicates.

### 2.4 Statistical analysis

Kruskal–Wallis test was used to determine the difference in each measurement. Bonferroni was used for the post hoc test. A comparison of the pressure of each group was performed by Bonferroni's post hoc method. To reconfirm the results, the data underwent analysis using GraphPad Prism 7.0. The analysis involved Dunn's multiple comparison test followed by Friedman's test, which is a non‐parametric statistical method. A *p*‐value < 0.05 was considered statistically significant.

## 3 RESULTS

### 3.1 Pressure by the method of hand infusion

In normal state, mean pressure of the 15‐G EZIO IO vascular needle was 13.96 mmHg and that of the 16‐G IV catheter was 86.88 mmHg. In the cephalic vein group, mean pressure of 16‐G IV catheterisation was 500.08 ± 118.42 mmHg. In the IO catheterisation state, mean pressure of the 15‐G EZIO IO needle group was 718.83 ± 93.09 mmHg, and mean pressure of the 16‐G IV catheter group was 605.06 ± 49.13 mmHg. The mean pressure of IO catheterisation using a 16‐G IV catheter was significantly lower than that using EZIO (*p* < 0.05).

### 3.2 Pressure by the methods of gravity drop and mechanical infusion

With the gravity drop method, mean pressure was 52.23 ± 13.45 mmHg in the cephalic vein group, 50.68 ± 11.89 mmHg in the 15‐G EZIO IO needle group and 52.23 ± 14.48 mmHg in 16‐G IV catheter group. With the mechanical infusion of 60 mL/kg, mean pressure was 98.26 ± 40.34 mmHg in the cephalic vein group, 128.25 ± 50.16 mmHg in the 15‐G EZIO IO needle group and 152.56 ± 67.23 mmHg in 16‐G IV catheter group (Table 1). The difference in pressure between the 15‐G EZIO needle group and the 16‐G IV catheter group was not significant.

**TABLE 1:** Median pressure of each group with gravity drop and mechanical infusion (IO: intraosseous; IV: intravenous) in six beagle dogs receiving IO catheterisation with a standard commercial catheter, compared to an EZIO cannula.

Group	Procedure	Median ± ranges (mmHg)
Gravity drop	16‐G IV catheter to IV for 5 min	52.23 ± 13.96
	15‐G EZIO to IO for 5 min	50.68 ± 11.89
	16‐G IV catheter to IO for 5 min	52.23 ± 14.48
Mechanical infusion	16‐G IV catheter to IV 60 mL/kg for 5 min	98.26 ± 40.34
	15‐G EZIO to IO 60 mL/kg for 5 min	128.25 ± 50.68
	16‐G IV catheter to IO 60 mL/kg for 5 min	152.56 ± 67.23

### 3.3 Procedure time and success rate

The difference in procedure time of the IO catheterisation between 15‐G EZIO IO needle catheterisation and 16‐G IV catheter IO catheterisation was 4.91 s (Table 2). The success rate was 83.33% with five out of six successful in both cases at the first attempts. In the case of unsuccessful 15‐G EZIO IO needle catheterisation, the catheter was inserted into the trochanteric fossa but inadvertently penetrated the lateral surface of the femoral body. Similarly, for the unsuccessful 16‐G IV catheter IO catheterisation, the catheter was directed into the trochanteric fossa but inadvertently penetrated near the distal femoral neck.

**TABLE 2:** Procedure time of IO catheterisation both 15‐G EZIO and 16‐G IV catheter.

Group	No. of individuals	Time (s)	Average (s)
15‐G EZIO to IO	1	13.28	11.84
	2^a^	12.31	
	3	11.65	
	4	11.82	
	5	11.54	
	6	10.41	
16‐G IV to IO	1	17.32	16.75
	2	16.82	
	3	16.49	
	4	17.05	
	5	16.27	
	6^a^	16.53	

^a^ Failed at the first attempt.

## 4 DISCUSSION

The present study was designed to compare the pressure through the 15‐G EZIO and normal 16‐G IV catheter stylet by applying them to IO catheterisation. However, we found no significant difference between the 15‐G EZIO and the commercial 16‐G IV catheter in IO catheterisation in perfusion via pressure. According to the results of the current study, IO catheterisation using an IV catheter stylet can be a substitute for the EZIO vascular system in clinical practice. Usually, IO catheter placement sites can be chosen from among the tibia, humerus or femur (Hammer et al., 2015). In this study, the trochanteric fossa of the femur was chosen as the insertion site under the length of a 16‐G IV catheter. The femur site works especially well for small breeds and pediatric patients. Since mobility is more challenging to control in dogs, compared to humans, we chose the femur site where the needle can be positioned as stably as possible. However, it is important to note that the stylet may be susceptible to breaking. Therefore, the practitioner should arrange additional caution to prevent stylet breakage during deployment and ensure its correct insertion. Sterilisation of the drill may not be ready in emergency status. Several ways can be used to keep in a sterile environment, such as wearing sterile gloves, draping the handle of a drill with sterile cohesive bandages and wiping a drill with alcohol cotton or gauze. To ensure aseptic conditions during the procedure, the drill was covered with a surgical drape and periodically cleansed using alcohol. The procedure time of 15‐G EZIO to IO was faster than that of 16‐G IV to IO. This was because a commercialised automatic injection system was used, and the 15‐G EZIO needle was also shorter and harder than the 16‐G IV catheter. In the current study, the success rate of using IV catheters for IO catheterisation at the femur was found to be comparable to that of EZIO. However, due to the limited number of studies, further trials and additional research are necessary to fully evaluate the retention and durability of IV catheters when employed for IO catheterisation. In addition, the variation in needle sizes used can have an impact on the study data. The 15‐G EZIO needle had an inner diameter of 1.372 mm, whereas the 16‐G IV needle had an inner diameter of 1.194 mm. This difference in diameter may result in variations when measuring pressures during the study.

## 5 CONCLUSION

The results of this study indicate the viability of IO catheterisation using a commercial IV catheter as an alternative method in emergencies, especially when rapid vascular access is necessary.

## AUTHOR CONTRIBUTIONS

**Janghwan Kim**: Conceptualisation; formal analysis; methodology; investigation; writing—original draft; writing—review and editing. **Minsu Kim**: Conceptualisation; funding acquisition; methodology; project administration; supervision; writing—review and editing. **Daesik Kim**: Conceptualisation; data curation; investigation; methodology; resources; validation. **Daeyoon Seo**: Data curation; investigation; methodology. **Sungsoo Lim**: Data curation; investigation; methodology. **Hansol Lee**: Data curation; investigation; methodology. **Taegyu Jung**: Data curation; investigation; methodology. **Bumgwan Namgoong**: Data curation; investigation; methodology. **Areum Choi**: Data curation; investigation; methodology.

## CONFLICT OF INTEREST STATEMENT

The authors report no conflicts of interest.

## ETHICS STATEMENT

The authors confirm that the ethical policies of the journal, as noted on the journal's author guidelines page, have been adhered to and the appropriate Ethical Review Committee approval has been received. This study was approved and received ethic clearance from Institutional Animal Care and Use Committee at Seoul National University (SNU‐230906‐7).

### PEER REVIEW

The peer review history for this article is available at https://www.webofscience.com/api/gateway/wos/peer-review/10.1002/vms3.1361.

## Data Availability

The data that support the findings of this study are available upon request from the corresponding author.
